# Enhancing treatment adherence in dialysis patients through digital health interventions: a systematic review and meta-analysis of randomized controlled trials

**DOI:** 10.1080/0886022X.2025.2482885

**Published:** 2025-03-26

**Authors:** Zhe Zhang, Xin-Ting Liang, Xu-Wei He, Xian Zhang, Rui Tang, Rui-Duo Fang, Jin-Zhu Li

**Affiliations:** a Department of the Six Health Care, The Second Medical Center and National Clinical Research Center for Geriatric Diseases, Chinese PLA General Hospital, Beijing, China; b Chinese PLA Medical School, Beijing, China

**Keywords:** Digital health interventions, adherence, systematic review, dialysis, telemedicine, artificial intelligence

## Abstract

**Objective:**

To systematically assess the efficacy of digital health interventions (DHIs) for improving treatment adherence among dialysis patients through a meta-analysis of randomized controlled trials (RCTs).

**Methods:**

Five databases were systematically searched from inception to April 2024. Meta-analyses were performed to calculate standardized mean differences (SMDs) and 95% confidence intervals (CIs) for adherence outcomes. Evidence quality was evaluated using the GRADE approach.

**Results:**

Seventeen RCTs involving 1,438 dialysis patients were analyzed. DHIs significantly improved overall adherence (SMD 1.88 [95% CI: 0.46–3.29]; 4 trials, low-certainty evidence). Specifically, DHIs demonstrated large improvements in medication adherence (SMD 1.45 [95% CI 0.38–2.52]; 4 trials, 300 patients; low-certainty evidence) and dialysis treatment adherence (SMD 1.88 [95% CI 0.46–3.29]; 4 trials, 245 patients; low-certainty evidence). Moderate improvements were observed in dietary adherence (SMD 0.58 [95% CI 0.25–0.91]; 4 trials, 344 patients; moderate-certainty evidence) and fluid management adherence (SMD −0.36 [95% CI −0.64 to −0.07]; 7 trials, 619 patients; moderate-certainty evidence).

**Conclusions:**

Digital health interventions effectively enhance multiple dimensions of treatment adherence in dialysis patients, underscoring their value for incorporation into routine clinical practice.

## Introduction

Chronic kidney disease (CKD) is a global public health concern, with millions of individuals worldwide progressing to end-stage renal disease (ESRD) requiring dialysis or transplantation [1–3]. Hemodialysis and peritoneal dialysis remain the most prevalent renal replacement therapies for ESRD patients. While these interventions are life-sustaining, they impose significant physical, psychological, and lifestyle burdens on patients [4,5]. Adherence to prescribed dialysis regimens, dietary restrictions, fluid management, and medication intake is critical for optimizing treatment outcomes and improving patient quality of life [6]. However, treatment adherence remains a significant challenge in this population [7].

Non-adherence in dialysis patients is multifactorial, involving complex interactions between individual patient factors, healthcare systems, and the chronic nature of the disease itself [8–10]. Poor adherence has been associated with increased morbidity, higher hospitalization rates, and decreased survival [11], underscoring the need for effective interventions to promote adherence. Traditional strategies to improve adherence, such as patient education and counseling, have shown limited success [12]. In recent years, the advent of digital health interventions (DHIs) has emerged as a promising approach to address this challenge [13–15]. Digital health interventions encompass a wide range of technologies, including mobile health applications, telemedicine, wearable devices, and other digital platforms that can facilitate patient engagement, self-monitoring, and communication with healthcare providers [16,17].

The potential of DHIs to enhance treatment adherence in dialysis patients is supported by several theoretical frameworks, including the Health Belief Model and the Technology Acceptance Model, which suggest that these technologies can positively influence patients’ health behaviors and attitudes [15,18]. However, the effectiveness of DHIs in this specific population remains a subject of ongoing investigation, with existing studies yielding heterogeneous results. Some research has demonstrated that DHIs can improve adherence by providing timely reminders, offering real-time feedback, and enabling personalized interventions [19–21]. Conversely, other studies have reported minimal or no significant impact [22,23], potentially due to variability in study designs, sample sizes, intervention types, and outcome measures.

Given the critical importance of adherence in the management of dialysis patients and the growing interest in DHIs as a tool to enhance adherence, a comprehensive synthesis of the current evidence is warranted [18,24]. To our knowledge, no systematic review and meta-analysis have yet been conducted to rigorously evaluate the effectiveness of DHIs on treatment adherence in dialysis patients. This systematic review and meta-analysis aim to fill this gap by assessing the impact of DHIs on adherence outcomes and providing evidence-based recommendations for clinical practice and future research.

## Methods

This systemic review was carried out following the methods of the Cochrane Handbook [25] for Systematic Reviews and reported in accordance with the Preferred Reporting Items for Systematic Review and Meta-Analyses (PRISMA) statement [26]. The protocol was published in the International Prospective Register of Systematic Reviews (PROSPERO): CRD42024566486.

### Information sources and search strategy

A search of PubMed, EMBASE, The Cochrane Central Register of Controlled Trials, Scopus, and Web of Science was performed in April 2024, unlimited by language or publication date. The search was designed and performed by an independent librarian; full details are available eTable 1 in the online supplementary. Three components of the search strategy (dialysis, digital health interventions, adherence) were formulated independently and subsequently integrated using database-specific truncation terms. Additionally, a hand citation search of included studies and relevant systematic reviews was performed to identify potentially eligible additional original papers. In cases where the full manuscript was not available, the author was contacted by e-mail.

### Study selection and data extraction

Two authors independently extracted data from the included studies following the guidelines of the Cochrane Handbook. Discrepancies were resolved through discussion as necessary. Randomized controlled trials (RCTs) (including quasi-randomized) investigating adherence in digital health intervention compared with non-digital intervention for dialysis patients were eligible for inclusion. Patients with end stage kidney disease undergoing dialysis were eligible. Digital health interventions [27,28] include ‘eHealth’ (information communication technologies for health), ‘mHealth’ (mobile-enabled digital health), and specific tools such as short messaging services (SMS) and electronic health records (EHR). The control group received routine dialysis treatment without digital health interventions. This systematic review primarily examines outcome measures related to treatment adherence. Based on available literature [29,30], dialysis adherence is classified into the following categories: total adherence, dialysis adherence, medication adherence, dietary adherence, and fluid management adherence.

We extracted the following data, as detailed in the characteristics of the included studies table: author, country of study, published year, participant characteristics (sample size, age, and gender), intervention details, and outcome measures related to dialysis treatment adherence. We defined short-term as less than three months, intermediate-term as six months to one year, and long-term as one year or longer. If sufficient studies are available, data from different time periods will be extracted for meta-analyses. Animal trials and non-English studies were excluded.

### Risk of bias assessment

Studies meeting the inclusion criteria were formally evaluated for risk of bias using the Cochrane risk of bias tool (version 2, ROB2) [31], assessing for the randomization process, intended interventions, missing outcome data, measurement of the outcome, and selection of the reported result. Each trial underwent assessment in these five bias domains, resulting in a summary risk-of bias score for each domain and an overall classification (low risk, some concerns, or high risk of bias). Two authors assessed each of the included studies and each potential source of bias was graded as high, low or unclear risk of bias. Two reviewers independently conducted an evaluation of the methodological quality of the original articles. Any disagreements were addressed through discussion or adjudication. The outcomes from these assessments were visualized and analyzed using an Excel RoB2 tool [31,32].

### Data analysis

The standardized mean difference (SMD) with 95% CIs was used to pool the results of individual trials for continuous outcomes measured using different scales. The MD and 95% CIs were used for pooling continuous outcomes measured using the same scale. When considered appropriate, we used a random effects model to pool for results of comparable groups of trials in a meta-analysis. If the SD or mean was not reported in the original article, where possible, it was calculated from the available data following the Cochrane guidelines. In accordance with Cohen [32], the effect sizes were interpreted as: large (≥0.8), moderate (0.5–0.8), small (0.2–0.5), and trivial (<0.2). Where published data were inadequate for meta-analysis, study authors were contacted. In addition, unpublished subgroup data or missing data were requested from the the corresponding authors. Statistical heterogeneity was assessed using the *I*^2^ statistic for each outcome, with classification as low (*I*^2^ < 25%), moderate (*I*^2^ = 25–50%), substantial (*I*^2^ = 50–75%), and considerable (*I*^2^ > 75%) [25]. We performed exploratory subgroup meta-analyses (if each subgroup included at least two studies) to assess potential sources of heterogeneity and assess the associations between variables and intervention effects. Sensitivity analyses were conducted by sequentially omitting individual studies to evaluate the robustness of the meta-analysis results. We also conducted a *post hoc* sensitivity analysis: to investigate the effects of bias on the results of the meta-analysis by excluding studies classified as having a high risk of bias. We assessed the presence of publication bias visually by funnel plot and formally by the Egger test [33]. All statistical analyses were performed using Review Manager (version 5.4.1) and Stata 12.0.

### Summary of findings tables

The quality of evidence for each individual meta-analysis was evaluated based on the Grading of Recommendations Assessment, Development, and Evaluation (GRADE) criteria [34]. Two reviewers conducted the assessment of evidence quality using the GRADE system, resolving any disagreements through discussion. Since only RCTs were included in the meta-analyses, each result was initially rated as high-certainty evidence. Detailed GRADE rating criteria are shown in Supplement eTable2.

## Results

### Study selection

**Figure 1.**
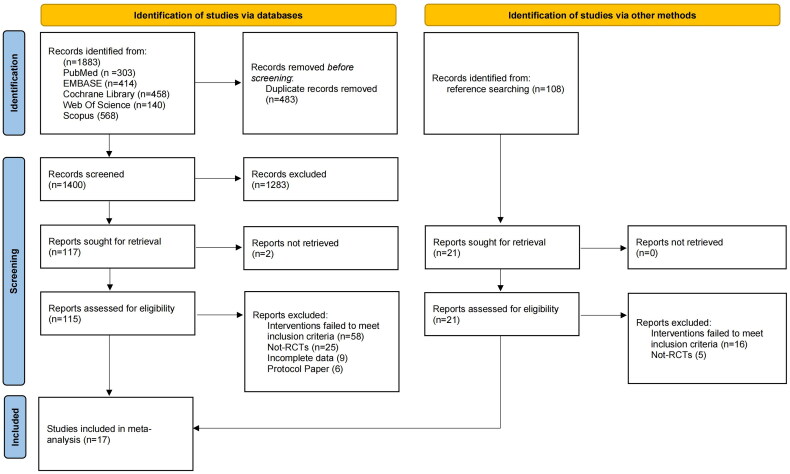
PRISMA flowchart describing study identification and selection process.

The search strategy identified 1,883 unique articles for screening. After screening, 115 full-text articles were retrieved, of which 98 articles were excluded for evaluation. Among the excluded articles, 58 were excluded for inappropriate interventions, 25 for incorrect study design, 6 for being protocol papers only, and 9 for incomplete data. A manual search did not identify any further articles. Finally, 17 articles were considered eligible for inclusion. The study selection process is summarized in the PRISMA flow diagram (Figure 1).

### Study characteristics

A total of 17 RCTs were identified, with 1.438 participants randomized with a mean age ranging from 27 years to 67 years. Descriptive characteristics of the 17 included trials are detailed in Table 1. Articles were published from 1981 to 2024, with 11 articles published in the last five years [20,21,23,35–41]. Three trials were conducted in Iran [20,22,38] and the USA [42–44] each, two trials were conducted in China [35,45], Korea [41,46], Australia [21,23] each, with the remainder in South Korea [36], India [37], Zahedan [39], Malaysia [40], and Germany [47]. The intervention period for the included trials was 5 to 24 weeks. Follow-up in most of the included trials was completed within three months, no studies had a follow-up longer than 12 months. There is significant variation in the elements of digital health interventions. Five trials conducted digital health interventions with app-based mHealth [23,35,36,38,39], four with SMS [20,21,42,46], two with video education [22,40], three with phone call [20,44,45], and three with technology-based self-monitoring [41,43,47]. Three trials assessed total adherence through two different adherence behavior measures using the End-Stage Renal Disease Adherence Questionnaire (ESRD-AQ) [20,38,39] and peritoneal dialysis-related health behavior [36].

**Table 1.** Subject demographics and baseline characteristics of the included studies.

Author(year), country	Sample characteristics N; Age		Description of intervention	Description of control	Outcomes measures
	Intervention group	Control group			
Zhang (2024), China	80;67.84(3.88)	80;67.39(3.92)	The hospital-community online management model: Patients kept in touch with medical staff through chronic disease management software and applets. 6 months	the outpatient follow-up management model 6 months	Medication adherence (GSES/MMAS-8)
Chae (2024), South Korea	27;45.48(11.60)	26;48.81(10.87)	Configuration of self-management mobile application applied three SCT factors to develop the SMA comprising of information regarding the disease and treatment, PD-related health behaviors, educational videos. 5 weeks	Usual care 5 weeks	PD-related health behavior
Vr (2023), India	55;53.42(12.32)	55;56.65(12.31)	a nurse-led brief advice, brief motivational intervention, telephonic booster intervention and daily morning reminder SMS 10 weeks	Usual care 10 weeks	Diet adherence (DDFQ); Fluid adherence (DDFQ)
Torabikhah (2023), Iran	35;45.26 (1.42)	35;46.74 (1.57)	The Di Care app consisted of different features, including educational materials, record medications for reminders and a self-test offline. 12 weeks	The educational materials were taught to the patients in person. three sessions per week, 4 weeks	Total adherence (ESRD-AQ); HD treatment adherence (ESRD-AQ); Medication adherence (ESRD-AQ); Fluid adherence (ESRD-AQ); Diet adherence (ESRD-AQ) Fluid adherence (IDWG).
Saadatifar (2023), Zahedan	40; 46.13(35.36)	40; 45.5(11.55)	mHealth training in five areas of treatment adherence using a smartphone application (My Dialysis) 3 months	Usual care 3 months	Total adherence (ESRD-AQ)
Teong (2022), Malaysia	33; 47.5(15.3)	33; 49.15(13.63)	the PMA carries six animated education videos and an interactive food database. 12 weeks	Usual care 12 weeks	Medication adherence (MMAS-4)
Jung (2021), Korea	22; 51.4(11.3)	28; 41.6(16.4)	The data sent from the remote patient monitoring for APD. Medical staff checked the APD information the next day and gave feedback based on yellow or red flags. 12 weeks	Traditional APD 12 weeks	Dialysis adherence, Dialysis accuracy, Dialysis adequacy
Dingwall (2021), Australia	62; 55.0(10.6)	33; 57.0(8.2)	using the AIMhi Stay Strong App comprising of two 20-min interviews, a text message or phone call 1 week following the initial treatment. 3 months	the questionnaires 3 months	Dialysis adherence (number of missing dialysis sessions)
Dawson (2021), Australia	87; 64.4(13.2)	43; 65.2(14.5)	usual care plus three text messages per week. 6 months	usual care 6 months	Diet adherence (Adherent with ≥3 renal dietary guidelines), Fluid adherence (IDWG / Meeting IDWG guidelines)
Arad (2021), Iran	33; 27.0(2.2)	33; 30.0(1.8)	received the patient education program, received telephonic calls twice a week and a text message every day. 3 months	usual care 3 months	Total adherence (ESRD-AQ), Dialysis adherence (ESRD-AQ), Medication adherence (ESRD-AQ), Fluid adherence (ESRD-AQ), Diet adherence (ESRD-AQ)
Som (2017), USA	9; 50.0(9.5)	10; 50.0(9.5)	The EpxDialysis intervention consisted of automated Short Message Service (SMS) texts or voice messages delivered to the patient’s preferred phone number three times per week. 8 weeks	Usual care 8 weeks	Dialysis adherence (number of missed sessions)
Sevick (2016), USA	93; 62.0(13.3)	85; 60.0(14.1)	Education and face-to-face meetings was delivered twice/week during the first 8 weeks, weekly in weeks 9–12, and every other week during weeks 13–16. 16 weeks	Education 16 weeks	Fluid adherence (Time-specific IDWG), Diet adherence (Time-specific dietary sodium intake).
Neumann (2013), Germany	60; 65.7(14.7)	49; 66.5(13.8)	Usual care and telemetric weight monitoring and once daily on days without dialysis. 3 months	Usual care 3 months	Fluid adherence (IDWG),
An (2011), Korea	19; 62.59(2.06)	20; 63.70(2.42)	patients were educated about their treatment regimen *via* e-mail 6 weeks	No e-mail education 6 weeks	Fluid adherence (IDWG), Diet adherence (Serum phosphate).
Wong (2010), China	49;62.4(/)	49;62.4(/)	Received both usual care and the intervention disease management programme. 6 weeks	Usual care 6 weeks	Dialysis adherence (CAPD days/ CAPD degree), Medication adherence (Medication days/ Medication degree), Fluid adherence (Fluid intake days/ Fluid intake degree), Diet adherence (Diet days/ Diet degree).
Baraz(2010), Iran	31; 34.85(9.51)	32; 34.85(9.51)	Video education through showing a video to each patient. They watched the 30-minute film twice a week. 2 months	Oral education 2 months	Fluid adherence (IDWG)
Cummings (1981), USA	27;54.8(13.75)	25;54.8(13.75)	Weekly Telephone Contacts. involved three activities: (1) gathering information from patients regarding problems they might be having in following their treatment instructions; (2) providing information (3) providing verbal support 11 min, once a week 6 weeks	Usual care 6 weeks	Fluid adherence (IDWG), Diet adherence (potassium levels).

NA: not available; PD: peritoneal dialysis; GSES: the General Self-Efficacy Scale; MMAS-8: the 8-item Morisky medication adherence scale; SCT: social cognitive theory; SMA: A self-management mobile application; DDFQ: dialysis diet and fluid non-adherence questionnaire; IDWG: Interdialytic weight gain; HD: Hemodialysis; APD: Automated peritoneal dialysis; ESRD-AQ: the End-Stage Renal Disease Adherence Questionnaire; CAPD: continuous ambulatory peritoneal dialysis.

### Risk-of-bias assessment

**Figure 2.**
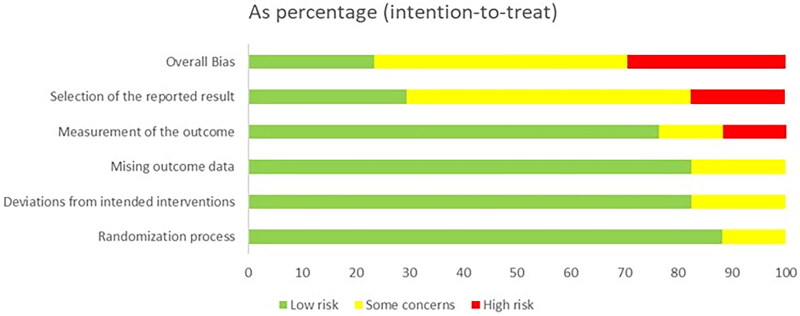
Risk of bias summary for all the included studies.

**Figure 3.**
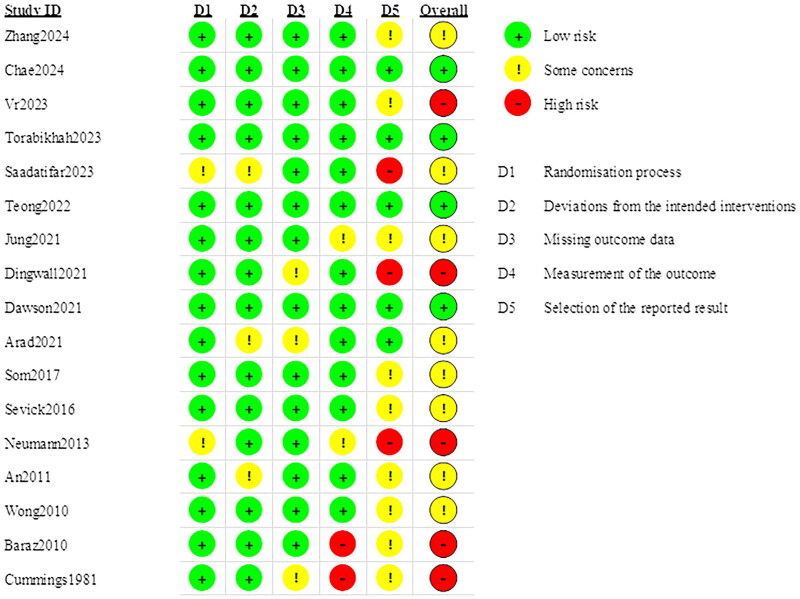
Summary of distribution of different biases.

Details of the risk of bias assessment are provided in Figures 2 and 3. The assessment resulted in a high risk of bias in 5 trial [22,23,37,44,47], some concerns of bias in 8 trials [20,35,39,41–43,45,46], and a low risk of bias in 4 trials [21,36,38,40]. Most of the trials were unclear or high risk of the selection of the reported result because of the lack a pre-registered protocol. Fifteen of the studies (88%) applied random sequence generation and allocation concealment.

### Effects of interventions

The GRADE evidence profile comparing the efficacy of digital versus non-digital health interventions for various outcomes is presented in Table 2.

**Table 2.** The GRADE summary of findings for the outcomes.

Digital compared to Non-digital for dialysis patients					
Outcomes	Anticipated absolute effects* (95% CI)		Relative effect (95% CI)	№ of participants (studies)	Certainty of the evidence (GRADE)
	Risk with Non-digital	Risk with Digital			
Total Adherence	–	SMD **1.88 higher** (0.46 higher to 3.29 higher)	–	269 (4 RCTs)	⨁⨁◯◯ Low^a,b^
Dialysis adherence	–	SMD **0.99 higher** (0.05 lower to 2.03 higher)	–	245 (4 RCTs)	⨁⨁◯◯ Low^a,b^
Medication adherence	–	SMD **1.45 higher** (0.38 higher to 2.52 higher)	–	300 (4 RCTs)	⨁⨁◯◯ Low^a,b^
Dietary adherence	–	SMD **0.58 higher** (0.25 higher to 0.91 higher)	–	344 (4 RCTs)	⨁⨁⨁◯ Moderate^b^
Fluid management adherence	–	SMD **0.36 lower** (0.64 lower to 0.07 lower)	–	619 (7 RCTs)	⨁⨁⨁◯ Moderate^c^
*The risk in the intervention group (and its 95% confidence interval) is based on the assumed risk in the comparison group and the **relative effect** of the intervention (and its 95% CI). **CI:** confidence interval; **MD:** mean difference; **OR:** odds ratio; **SMD:** standardized mean difference					
**GRADE Working Group grades of evidence** **High certainty:** we are very confident that the true effect lies close to that of the estimate of the effect. **Moderate certainty:** we are moderately confident in the effect estimate: the true effect is likely to be close to the estimate of the effect, but there is a possibility that it is substantially different. **Low certainty:** our confidence in the effect estimate is limited: the true effect may be substantially different from the estimate of the effect. **Very low certainty:** we have very little confidence in the effect estimate: the true effect is likely to be substantially different from the estimate of effect.					

^a^Evidence of serious inconsistency (heterogeneity in the *I*^2^ test >75%).

^b^The limited number of participants.

^c^The 95% CI spanned 0.

### Total adherence

**Figure 4.**
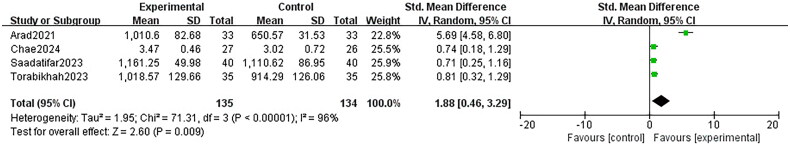
Forest plot for the total adherence.

There was a large improvement in total adherence compared with non-digital health interventions at short-term (SMD 1.88 [95% CI 0.46–3.29]; four trials, 269 patients; *I*^2^ 96%; GRADE: low) (Figure 4). However, after the exclusion of the study [20], the heterogeneity diminished significantly (*I*^2^ = 0%), and the outcomes remained significant (SMD 0.75 [95% CI 0.46–1.03]).

### Dialysis adherence

**Figure 5.**
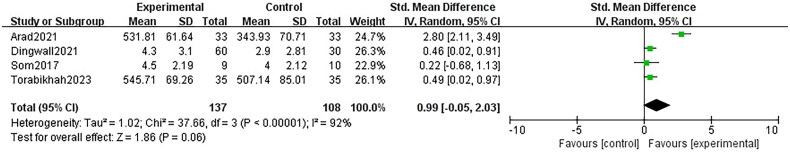
Forest plot for the dialysis adherence.

There was a large improvement in dialysis adherence compared with non-digital health interventions at short-term (SMD 0.99 [95% CI −0.05 to 2.03]; four trials, 245 patients; *I*^2^ 92%; GRADE: low) (Figure 5). However, statistical analysis revealed no significant difference (*p* = .06). After the exclusion of the study [20], the heterogeneity disappeared (*I*^2^ = 0%) with a significantly improvement (SMD 0.45 [95% CI 0.14–0.75]).

### Medication adherence

**Figure 6.**
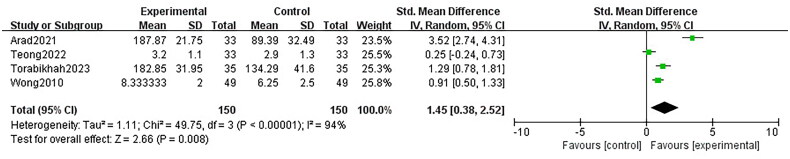
Forest plot for the medication adherence.

There was a large improvement in medication adherence ­compared with non-digital health interventions at short-term (SMD 1.45 [95% CI 0.38–2.52]; four trials, 300 patients; *I*^2^ 94%; GRADE: low) (Figure 6). Also, after the exclusion of the study [20], the heterogeneity diminished significantly (*I*^2^ = 77%), and the outcomes remained significant (SMD 0.81 [95% CI 0.24–1.39]).

### Dietary adherence

**Figure 7.**
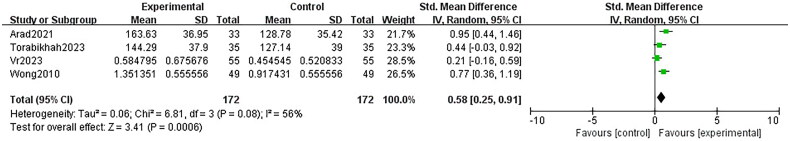
Forest plot for the dietary adherence.

There was a moderate improvement in dietary adherence compared with non-digital health interventions at short-term (SMD 0.58 [95% CI 0.25–0.91]; four trials, 344 patients; *I*^2^ 56%; GRADE: moderate) (Figure 7).

### Fluid management adherence

**Figure 8.**
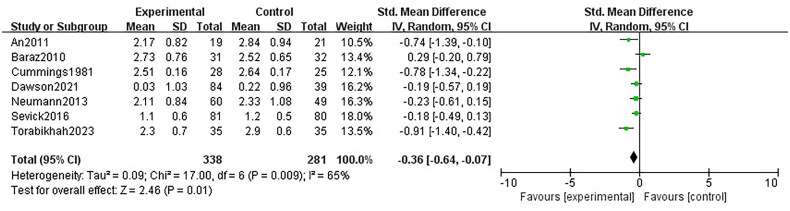
Forest plot for the fluid management adherence.

There was a small improvement in fluid management adherence compared with non-digital health interventions at short-term (SMD −0.36 [95% CI −0.64 to −0.07]; seven trials, 619 patients; *I*^2^ 65%; GRADE: moderate) (Figure 8).

### Publication bias and sensitivity analyses

Because of the small number of included studies, we only performed publication bias for fluid management adherence (*n* = 7). Visual inspection of funnel plots and the Egger test did not indicate publication bias (*p* = .255) (eFigure 1 in the Supplement). Two sensitivity analyses were performed: estimation of sensitivity excluding each study, estimation of sensitivity excluding the studies at high risk of bias. In the sensitivity analyses, only the meta-analysis result of dialysis adherence became more insignificant (1.18 [−0.40, 2.76], *p* = .14) (eFigure 2 in the Supplement) after excluding one study at high risk (Dingwall 2021). Nevertheless, the consistency of results for all other meta-analyses in the two sensitivity analyses supports the robustness of the findings.

### Subgroup analyses

Subgroup analyses by digital health intervention type (mobile app vs telephone and/or SMS vs other digital platforms) for total adherence, medication adherence, dietary adherence, and fluid management adherence were not done owing to only one study being included in one or more subgroups. Subgroup analysis showed no difference in dialysis adherence (1.53, 95% CI −1.00 to 4.05) in telephone and/or SMS based interventions versus a significant improvement (0.48, 95% CI 0.15–0.80) in the mobile app-based intervention (eFigure 3 in the Supplement).

Subgroup analyses by control group type (usual care vs active control) for total adherence, medication adherence, and dietary adherence were not done owing to only one study being included in one or more subgroups. Subgroup analysis showed no difference in dialysis adherence (1.53, 95% CI −1.00 to 4.05) in usual care controls versus a significant enhancement (0.48, 95% CI 0.15–0.80) in active controls (eFigure 4 in the Supplement). The results of this subgroup analysis were the same as the previous subgroup analysis for dialysis adherence based on the digital health intervention. Theoretically, between-group differences should be smaller in the subgroups of active controls, but this subgroup analysis revealed the opposite result. This suggests that the type of digital health intervention exerts a more substantial influence on meta-analysis results than the control group type. Subgroup analysis showed no difference in fluid management adherence (−0.26, 95% CI −0.86 to 0.34) in active care controls versus significant improvement (−0.41, 95% CI −0.70 to −0.11) in usual care controls (eFigure 5 in the Supplement).

## Discussion

The findings of our systematic review and meta-analysis indicate that DHIs offer small to large improvements in various aspects of treatment adherence among dialysis patients with low- to moderate-quality evidence. Specifically, we observed large enhancements in total adherence, dialysis adherence, and medication adherence, albeit with low-quality GRADE evidence. The improvements observed in dietary adherence and fluid management adherence were moderate and small, respectively, both supported by moderate-quality GRADE evidence. However, due to the small number of included trials, results need to be interpreted with caution.

This systematic review and meta-analysis have illuminated the substantial potential of digital health interventions to enhance treatment adherence among dialysis patients. Specifically, we observed large effect sizes in total adherence, indicating that digital tools can effectively motivate and empower patients to engage more actively in their treatment regimens. However, the moderate and small improvements in dietary and fluid management adherence raise critical questions about the specific barriers patients face in these areas. It suggests that while technology can facilitate general adherence, dietary and fluid management may require more specialized interventions that provide direct guidance and support.

The results of the meta-analyses regarding total adherence, dialysis adherence, and medication adherence exhibited substantial heterogeneity. The heterogeneity observed in total and dialysis adherence may be partially attributed to the study by Arad et al. [20]. Upon exclusion of this study, heterogeneity was significantly diminished, resulting in *I*^2^ = 0%. A thorough examination of the study characteristics revealed that Arad et al.’s research [20] was the only one with an average participant age of 30 years or younger, suggesting that younger individuals may demonstrate greater adaptability and responsiveness to digital health interventions. In contrast, the elevated heterogeneity in medication adherence can be traced to multiple factors. Although the exclusion of Arad et al. [20] decreased the heterogeneity to *I*^2^ = 77%, it remained significantly heterogeneous. Furthermore, several studies within the meta-analysis employed varying scales to assess medication adherence, and the duration of interventions spanned from 6 to 24 weeks, contributing to the unavoidable clinical heterogeneity observed.

## Comparison with other studies

This is the first review to directly compare the efficacy of digital and non-digital health interventions for people with dialysis. Our results align with existing literature on digital health interventions across various chronic conditions [48,49]. This suggests a broader trend where digital health tools play a vital role in enhancing adherence across different chronic diseases. Conversely, some studies, like Murali et al. [12], have reported negligible improvements in treatment adherence among people with end stage kidney disease on dialysis using various adherence enhancement strategies. This discrepancy emphasizes the need for tailored interventions that consider the unique dietary restrictions and fluid management challenges inherent in dialysis care [50]. The differences in study outcomes may stem from variations in intervention design, duration, and patient populations, further highlighting the importance of context in evaluating the effectiveness of digital health tools.

## Limitations

While our review provides valuable insights, several limitations must be acknowledged. The low GRADE evidence for total, dialysis, and medication adherence indicates that the findings, while promising, should be interpreted cautiously. The variability in study designs, sample sizes, and types of interventions included in the meta-analysis may have influenced the overall effect size and contributed to heterogeneity. Additionally, many studies relied on self-reported measures of adherence, which are prone to bias [51]. Patients may overestimate their adherence due to social desirability, leading to inflated results that do not accurately reflect true behavior. The reliance on self-reported data, coupled with the variability in how adherence is measured, complicates direct comparisons across studies. Standardization of outcome definitions and measures is another area for improvement. The included studies used different definitions and measures of adherence, which created challenges in combining and interpreting results. Future reviews should presuppose standardized outcome definitions and provide clear criteria for merging different adherence measures. Separate analyses of different measurement tools may also be needed. Furthermore, the included studies primarily focused on urban populations, which may limit the generalizability of our findings. Rural or underserved populations may experience distinct barriers to engagement with digital health interventions, such as limited internet access or lack of technological literacy [52]. Future research should prioritize the inclusion of diverse populations to enhance the applicability of findings across different demographic groups.

## Future considerations

Future research should aim to address the identified limitations and explore several key areas. Longitudinal studies evaluating the long-term impacts of digital health interventions on adherence are critical. Understanding whether initial improvements in adherence can be sustained over time will provide deeper insights for healthcare providers and inform best practices in digital health implementation. Furthermore, an investigation into the efficacy of diverse digital health interventions, including interactive applications, telehealth consultations, and wearable technologies, could provide a more comprehensive understanding of the optimal modalities for different aspects of treatment adherence [53]. Tailoring interventions to target specific adherence challenges, such as dietary restrictions and fluid management, may enhance their effectiveness. This could involve developing user-friendly applications that offer personalized meal plans, fluid tracking, and real-time feedback on dietary choices [54]. Collaboration among nephrologists, dietitians, and digital health developers is essential to create comprehensive intervention strategies that meet the multifaceted needs of dialysis patients. Incorporating patient feedback during the design and implementation phases will ensure that digital health tools are user-centered, addressing real-world challenges faced by patients. Moreover, educational components that empower patients to understand their conditions and the importance of adherence may foster a sense of ownership and motivation [55].

## Conclusion

In conclusion, this systematic review and meta-analysis underscore the promising potential of digital health interventions to enhance treatment adherence among dialysis patients. Despite the current low to moderate quality of evidence, the substantial improvements observed in total, dialysis, and medication adherence are encouraging and warrant further exploration. As the healthcare landscape evolves in the digital age, nephrology practitioners must leverage these technological advancements to foster patient engagement and adherence. Future research should focus on establishing the long-term effectiveness of these interventions, exploring diverse populations, and refining strategies to address specific adherence challenges. By taking these steps, we can contribute to improved clinical outcomes and a better quality of life for dialysis patients worldwide.
